# Development of erianin-loaded dendritic mesoporous silica nanospheres with pro-apoptotic effects and enhanced topical delivery

**DOI:** 10.1186/s12951-020-00608-3

**Published:** 2020-03-30

**Authors:** Canlong Mo, Lulu Lu, Danyang Liu, Kun Wei

**Affiliations:** 1 School of Biology and Biological Engineering, South China University of Technology, Guangzhou, 510006 China; 2 Drug Research Institute, Guangzhou Baiyunshan Tianxin Pharmaceutical Co., Ltd, Guangzhou, 510006 China

**Keywords:** Skin delivery, Apoptosis, Erianin

## Abstract

**Background:**

Psoriasis is a malignant skin disease characterized as keratinocyte hyperproliferation and aberrant differentiation. Our previous work reported that a bibenzyl compound, erianin, has a potent inhibitory effect on keratinocyte proliferation. To improve its poor water-solubility, increase anti- proliferation activity, and enhance the skin delivery, erianin loaded dendritic mesoporous silica nanospheres (E/DMSNs) were employed.

**Results:**

In this work, DMSNs with pore size of 3.5 nm (DMSN_1_) and 4.6 nm (DMSN_2_) were fabricated and E/DMSNs showed pore-size-dependent, significantly stronger anti-proliferative and pro-apoptotic effect than free erianin on human immortalized keratinocyte (HaCaT) cells, resulting from higher cellular uptake efficiency. In addition, compared to free erianin, treatment with E/DMSNs was more effective in reducing mitochondrial membrane potential and increasing cytoplasmic calcium levels, which were accompanied by regulation of mitochondria and endoplasmic reticulum stress (ERS) pathway. Porcine skin was utilized in the ex vivo accumulation and permeation studies, and the results indicated higher drug retention and less drug penetration in the skin when administered as the E/DMSNs-loaded hydrogel compared to the erianin-loaded hydrogel.

Conlusions

This work not only illustrated the further mechanisms of erianin in anti-proliferation of HaCaT cells but also offer a strategy to enhance the efficiency of erianin and the capacity of skin delivery through the DMSNs drug delivery systems.

## Background

Psoriasis, a chronic immunoinflammatory skin disease, affects about 1–3% of individuals worldwide and significantly decreases the patient’s quality of life. Although the exact pathophysiology of psoriasis is not yet fully clarified, the keratinocyte hyperproliferation, abnormal keratinocyte differentiation and prominent infiltration of immunocytes are considered to be the primary factors that facilitate its development [1]. Consequently, inhibition of the extreme proliferation of keratinocytes has proved to be one of the effective choices for psoriasis treatment.

Apoptosis, a process of programmed cell suicide, is generally involved by the extrinsic pathway, the intrinsic pathway, and ERS [2]. The intrinsic pathway is gated through proteins of BCL-2 family, which regulate the production of specific caspase-activating proteins from damaged mitochondria [3]. The extrinsic pathway is caused by the engagement of cell surface death receptors, which convert signals from extracellular stimuli to the intracellular caspase machinery [4]. The ERS is triggered by a variety of extracellular stimuli such as heat shock, ischemia, and hypoxia, which leads to cell death under excessive ERS [5]. Many studies have shown that inducing keratinocyte apoptosis are been considered to be the therapeutic strategies for psoriasis [6, 7].

Erianin, a low molecular weight natural product extracted from *Dendrobium chrysotoxum Lindl*, has been shown therapeutic potential to suppress tumor growth and angiogenesis in vivo and in vitro [8, 9]. Recently, we have demonstrated that erianin exhibited growth suppression and apoptosis in HaCaT cells [10]. However, whether erianin induced HaCaT cell apoptosis via mitochondrial and ERS signaling pathways remains unclear. Although erianin has demonstrated a direct inhibitory effect on keratinocyte proliferation, its poor water solubility and low penetration activity across the skin limited its in vivo application. In addition, topical administration is the preferable administration for psoriasis treatment which has more therapeutic effects, lower drug systemic toxic effects, and higher patient compliance [11]. Hence, drug-delivery systems to improve erianin delivery are indispensably needed in the topical therapy of psoriasis.

Since the early 1980s, nanotechnology has been applied in the topical therapy of skin diseases [12]. Owing to the high affinity to skin barriers and good biocompatibility in loading lipophilic drugs, lipid-based and polymeric nanoparticles have been used widely in the topical therapy of skin diseases [13, 14]. But there were still some problems like highly hydrophobic property, worse biodegradability, and slow drug release in polymeric nanoparticles [15]. Recently, mesoporous silica nanoparticles have drawn the attention as carrier system for the topical delivery of active chemical compounds because of their unique properties such as extremely high surface area, ordered porosity, and tunable pore volume [16, 17]. Therefore development of mesoporous silica nanoparticles drug-delivery systems offer an alternative administration route for the therapy of cutaneous disorders.

**Scheme 1:**
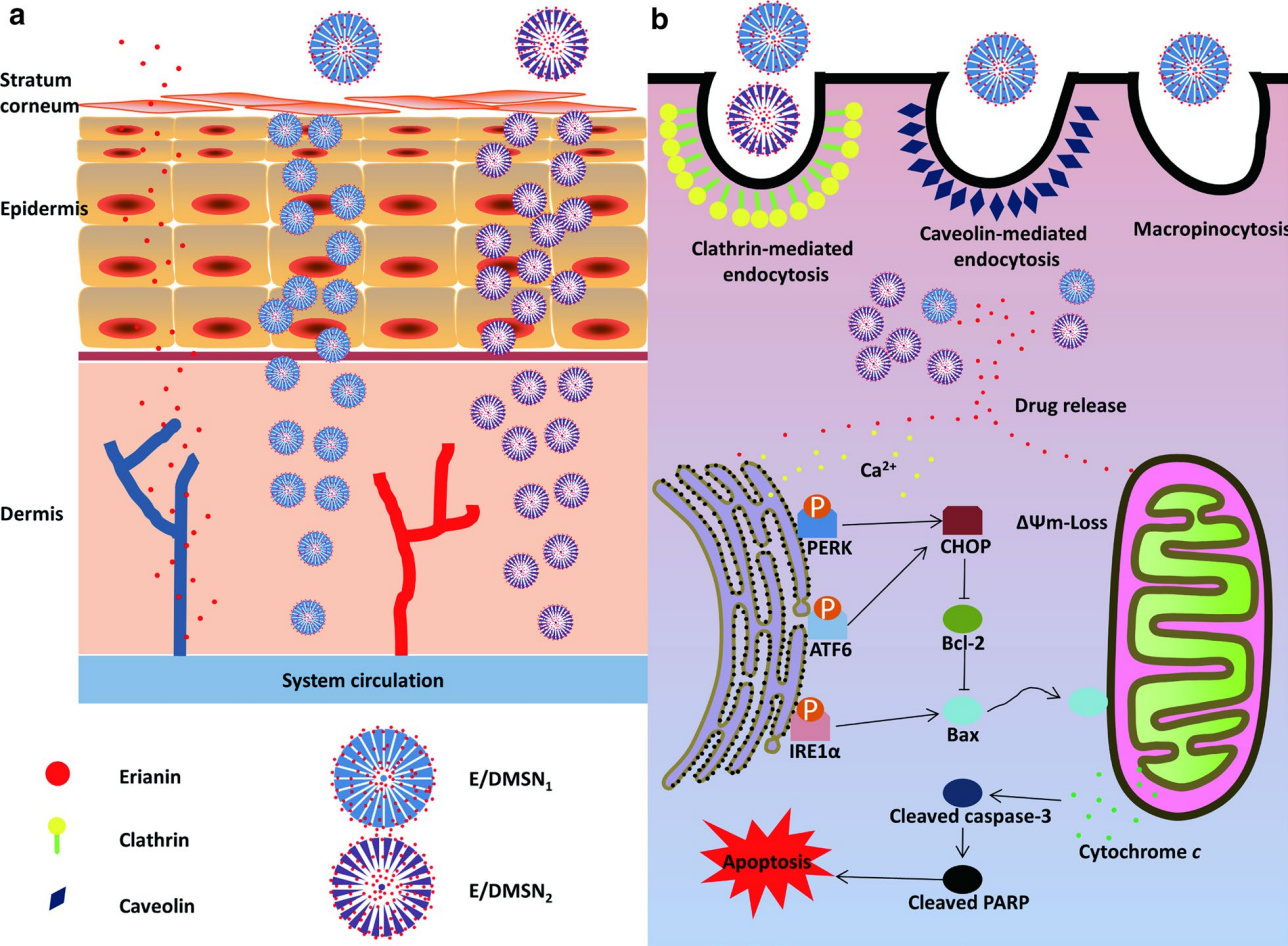
Schematic illustration of topical application of E/DMSNs to skin (**a**), and their mechanisms on pro-apoptotic effect in HaCaT cells (**b**)

Different pore sizes of MSNs showed different effects on drug loading efficacy [18], drug release behavior [19], and cellular uptake rate [20]. To the best of our knowledge, there has been no research on the different pore sizes of DMSNs as a skin delivery system and against HaCaT cells with the exploration of physiological mechanisms. In the present study, two different pore sizes of E/DMSNs were investigated for in vitro drug release, cellular uptake behavior, endocytosis mechanism, and ex vivo permeation study on porcine skin. Moreover, the effect on HaCaT cell proliferation, apoptosis, and its possible mechanisms were tested, compared to that of free erianin (Scheme 1).

## Materials and methods

### Materials

Cetyltrimethylammonium chloride (CTAC) solution (25 wt % in H_2_O), triethanolamine (TEA), and 1-octadecene were purchased from Sigma-Aldrich (St. Louis, MO, USA). Tetraethyl orthosilicate (TEOS), cyclohexane, 3-aminopropyltriethoxysilane (APTES), and fluorescein isothiocyanate (FITC) were purchased from Aladdin Reagent (Shanghai, China). Erianin (≥ 98% purity) was purchased from Chengdu Herbpurify (Chengdu, China). HaCaT cell line was purchased from Kunming Cell Bank of the Chinese Academy of Sciences (Kunming, China). Dulbecco modified Eagle medium (DMEM), fetal bovine serum (FBS), penicillin, and streptomycin were purchased from Thermo Scientific (WI, USA). Chlorpromazine, genistein, and amiloride were purchased from Solarbio (Beijing, China). 3-(4,5-dimethylthiazol-2-yl)-2,5-dipheny-ltetrazolium bromide (MTT) was purchased from Biofroxx (Einhausen, German). Dimethyl sulfoxide (DMSO) was purchased from MP Biomedicals (OH, USA). Annexin V-FITC/propidium iodide (PI) staining kit was purchased from BestBio (Shanghai, China). Fluorescent mitochondrial probe JC-1 and fluorescent calcium ion probe Fluo-4 AM were purchased from Beyotime Biotechnology (Shanghai, China). BCA protein assay kit was purchased from Sangon Biotech (Shanghai, China). Antibodies against Bcl-2, Bax, cytochrome *c*, cleaved caspase-3, cleaved poly (ADP-ribose) polymerase (PARP), activating transcription factor 6 (ATF6), and inositol-requiring enzyme 1α (IRE1α) were purchased from Cell Signal Technology (Boston, USA). Protein kinase RNA-like ER kinase (PERK) and C/EBP homologous protein (CHOP) were purchased from Affinity Biosciences (OH, USA). Carbopol 974 was purchased from Xinhenglong Technology (Wuhan, China). All chemicals obtained were used without additional purification.

## Synthesis of DMSNs

DMSNs were synthesized according to the previously reported method [21]. Briefly, to obtain DMSN_1_, 48 mL of (25 wt %) CTAC solution and 0.36 g of TEA were dissolved in 72 mL of water and stirred mildly at 60 °C for 1 h in a 250-mL round bottom flask, then 40 mL of (20 v/v %) TEOS in 1-octadecene was added dropwise. A prismatic Teflon-coated stirring bar with a length of 4 cm was employed, and the resulting mixture kept stirring for 10 h under the stirring rate of 150 rpm. The products were collected by centrifugation and washed with ethanol several times to remove the residual reactants. Then the collected products dried in vacuum overnight and were calcinated at 550 °C for 5 h, resulting in the final formation of DMSN_1_. The synthesis of DMSN_2_ followed the same procedure as reported for DMSN_1_ with a little change: after TEA addition and the stabilization of the temperature, 40 mL of (10 v/v %) TEOS in cyclohexane was added and kept stirring for 12 h.

## Drug loading

Erianin loading was performed using a rotary evaporation technique according to the previously reported method [22]. 100 mg of DMSNs was placed in a rotary evaporation flask followed by addition of 5 ml of (30 mg/mL) erianin in methanol and sonicated for 30 min. The solvent was slowly evaporated using a rotary evaporator at 50 °C for 2 h to obtain the dry powder. Then the collected products dried in vacuum overnight, resulting in the final formation of E/DMSNs.

The concentration of erianin was measured by a Waters Acquity H class UPLC system. The UPLC analysis was performed by an apparatus consisting of a quaternary pump and a TUV detector (Waters, Milford, MA, USA). The chromatographic separation was performed using an Acquity UPLC BEH C18 column (2.1 × 100 mm; 1.7 μm, Waters) at 35 °C. The mobile phase was acetonitrile and water eluting in a gradient mode at a flow rate of 0.3 mL/min. The detection wavelength was set at 232 nm. Data processing was determined using Empower 3 software (Waters, Milford, MA, USA).

The quantitative loading of erianin was determined by dispersing 10 mg of each complex in 1 mL of methanol with sonicating for 30 min. The mixture was then centrifuged and filtered with a 0.22 μm filter before analyzing by the UPLC. The drug loading capacity (LC%) was calculated according to the following equation:

1 $$\text{LC}\% = \left( {{\text{Amount of drug in the complex}}/{\text{Total amount of the complex}}} \right) \times 100\%$$

### Characterization

Transmission electron microscopy (TEM) analysis was performed on a FEI TECNAI G2 F20 electron microscope (FEI, USA). The size distribution and zeta potential of nanospheres were measured by dynamic light scattering (DLS) using a Zetasizer Nano ZS (Malvern, UK). The average pore size and specific surface areas (SSA) were determined by nitrogen adsorption–desorption isotherms using an Autosorb-iQ Automated Gas Sorption Analyzer (Quantachrome, USA). Another method to quantify the loading of erianin was determined by thermogravimetric analysis (TGA) using a 449C simultaneous thermal analyzer (Netzsch, Germany). Fourier transform infrared (FTIR) spectra were obtained by a Nicolet CCR-1 spectrometer (Thermo, USA). Powder X-ray diffractograms (XRD) were collected by an Empyrean X-ray diffractometer (PANalytical, Netherlands).

## In vitro release

The drug release study of erianin was performed using (8,000–14,000 Molecular Weight Cut Off) dialysis membrane (Shanghai yuanye Bio-Technology, China) at the temperature 37 °C. The phosphate buffered solution (PBS, pH 7.4) was chosen as donor and receiving phase. At predetermined time intervals (1, 2, 3, 4, 5, 6, 7, 8, 11, 14, 24, 36, 48 h), an aliquot of the receiving phase was withdrawn and immediately replaced with an equal volume of PBS. The collected samples were then analyzed for erianin content by UPLC.

## Cell culture

HaCaT cells were cultured in DMEM supplemented with 10% FBS, 100 U/mL penicillin and 100 µg/mL streptomycin at 37 °C in a humidified incubator with 5% CO_2_.

## Cellular uptake experiment

Fluorescence labeling of DMSNs was synthesized according to the previously described procedure with modifications [23]. Firstly, 200 mg of DMSNs were dispersed in 10 mL of ethanol. Then, 200 μL of APTES was added, and the solution kept stirring for 12 h at 70 °C under the stirring rate of 500 rpm. The products were collected by centrifuging and washed three times with ethanol, and dried in vacuum at 50 °C. Then, 500 mg of these products were dispersed in 10 mL of ethanol, and 4.0 mg of FITC was added, and the solution kept stirring for 24 h at room temperature under the stirring rate of 500 rpm. The products were separated by centrifugation and washed three times with ethanol. The resulting products were obtained after drying in vacuum at 50 °C.

To show the cellular uptake efficacy of DMSNs, flow cytometry study was employed. HaCaT cells were seeded in 6 well plates at a density of 2 × 10^5^ /mL and incubated for 24 h. Then the cells were treated with 1 μg of FITC-labeled DMSNs for up to 24 h. At predetermined time point (0.25, 0.5, 1, 2, 4, 8, 12, 18, 24 h), the cells were washed three times with PBS and trypsinized. Then the cells were collected and washed three times with PBS, and analyzed by a flow cytometer Accuri C6 (BD, USA).

To investigate the endocytosis mechanism involved in the uptake process, cells were pre-treated with endocytic inhibitors including chlorpromazine (100 μM), genistein (200 μM), and amiloride (200 µM) for 30 min. Then the cells were treated with 1 μg of FITC-labeled DMSNs for 6 h. In a control group, cells were not treated with the inhibitors prior to nanoparticle treatment. Cellular uptakes of nanoparticles were quantitated by flow cytometer as described above.

## Cell proliferation assay

HaCaT cells were seeded in 96 well plates at a density of 2 × 10^4^ cells/well and cultured for 24 h. Then the cells were treated with 30 nM erianin and E/DMSNs equivalent to 30 nM erianin for 24 h. Cells were then treated with 10 µL of MTT reagent (5 mg/mL) for 2 h at 37 °C. 200 µL of DMSO was added to each well and the absorbance at 570 nm was determined by a microplate reader (PerkinElmer, USA).

## Annexin V/PI staining assay

Apoptotic cells were determined by Annexin V/PI staining assay. HaCaT cells were plated into 6 well plates at a density of 2 × 10^5^/mL and cultured for 24 h. Then the cells were treated with 30 nM erianin and E/DMSNs equivalent to 30 nM erianin for 24 h. After that, cells were collected, washed 3 times with cold PBS and incubated with 5 µL Annexin V-FITC solution for 15 min in the dark. The cell suspensions were stained with 5 µL PI solution for 5 min. Finally, 400 µL Annexin V binding solution was added to cell suspensions, and then analyzed by flow cytometry. The apoptosis rate was calculated by adding the percentage of early and late apoptosis rates.

## Mitochondrial membrane potential detection

Mitochondrial membrane potential was measured by fluorescent mitochondrial probe JC-1. Briefly, after treatment with 30 nM erianin and E/DMSNs equivalent to 30 nM erianin for 24 h, cells were collected and incubated with 1 mL of JC-1 working solution at 37 °C for 20 min. The cell suspensions were washed thoroughly and resuspend with dye buffer solution, and then analyzed by flow cytometry.

## Cytoplasmic calcium concentration detection

Cytoplasmic calcium levels were detected by Fluo-4 AM probe. Briefly, after treatment with 30 nM erianin and E/DMSNs equivalent to 30 nM erianin for 24 h, cells were collected and incubated with 1 mL of Fluo-4 AM (2 µM) in PBS at 37 °C for 30 min. The cell suspensions were washed thoroughly and resuspend with PBS, and then analyzed by flow cytometry.

### Western blot analysis

Total proteins were extracted using ice-cold radio-immunoprecipitation assay (RIPA) buffer. Protein estimation was detected by a BCA protein assay kit. Protein samples (50 µg) were separated by SDS-PAGE and then blotted onto a poly (vinylidene fluoride) (PVDF) membrane. The membranes were blocked with 5% non-fat milk in tris-buffered saline and Tween 20 (TBST) buffer for 1 h at room temperature. Then the membranes were incubated with specific primary antibody at a dilution of 1:1000 at 4 °C overnight. Next, the membranes were washed with TBST buffer and probed with secondary antibodies at a 1:10,000 dilution at room temperature for 1 h. The membranes were placed on a visualization strip by the Chemiluminescence Kit (Millipore, USA) with the Amersham Imager 600 imager (GE Healthcare Life Science, USA).

## In vitro skin permeation studies

For the in vitro skin permeation studies, Carbopol gel was chosen as the hydrogel according to the previously report [24]. 100 mg of Carbopol was added to 10 g of water and kept slow constant stirring for 24 h. The gel was adjusted the pH value to 6 by adding dropwise of TEA after adequate swelling, resulting in the final formation of 1% (w/v) Carbopol gel. E/DMSNs or erianin were added into the Carbopol gel and stirred for 24 h to mix uniformly.

The skin permeation of erianin and E/DMSNs-loaded gel was performed through porcine skin by using vertical Franz cells with available diffusion area of 2.8 cm^2^. Pig ears skin freshly obtained from a local slaughterhouse and served as a model barrier. The skin was mounted between the donor and the receptor chambers with the stratum corneum facing up. 3 g of E/DMSNs-loaded gel or erianin-loaded gel with equivalent amount of drug (10 mg) was placed on the epidermal surface of skin as the donor chamber. The receptor chamber was filled with 6.5 mL of PBS/ethanol (80/20, v/v) and was continuously stirred at 37 °C for 24 h. Afterwards, the effective diffusion area of skin was washed with PBS thoroughly. The skin was then cut into small pieces and kept stirring for 24 h at room temperature in the addition of 3 mL of methanol. The samples were sonicated for 30 min and measured with UPLC after centrifugation. The results were expressed as erianin amount *versus* skin diffusion area (µg/cm^2^).

### Statistical analysis

Statistical analysis was calculated using GraphPad Prism (GraphPad Software 6.0, USA). All data were duplicated from three independent experiments, and the results are expressed as the mean ± standard deviation (SD). Student’s *t*-test was used to analyze the significant differences between two samples, while one-way ANOVA test was employed to substantiate statistical differences between groups. Statistical significance is expressed by *p* < 0.05.

## Results and discussion

### Characterization of E/DMSNs

**Fig. 1:**
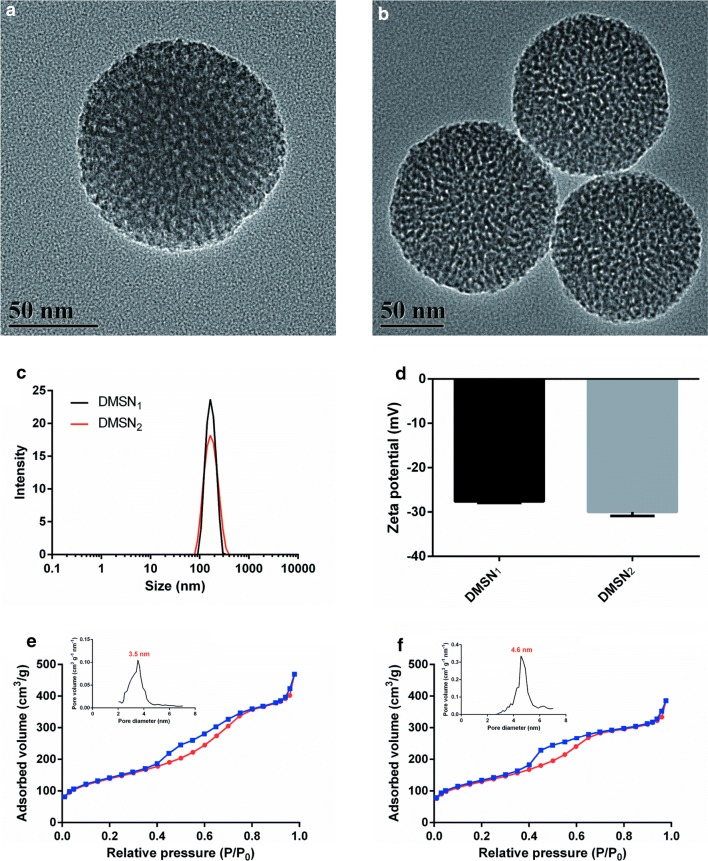
Characterization of DMSNs. TEM images of **a** DMSN_1_ and **b** DMSN_2_. **c** The size distribution of DMSNs. **d** Zeta potential of DMSNs. Ntrogen adsorption–desorption isotherm and corresponding pore size distributions of (**e**) DMSN_1_ and **f** DMSN_2_. The values are expressed as means ± SD (n = 3)

The TEM images of DMSN_1_ and DMSN_2_, revealed the presence of uniform nanospheres, characterized by regular and ordered channels (Fig. 1a, b, and Additional file 1: Fig. S1). DLS measurements showed a mean size of 164 nm and 161 nm for DMSN_1_ and DMSN_2_, respectively (Fig. 1c). The zeta potential values of −27.6 mV and −30.3 mV for DMSN_1_ and DMSN_2_, respectively (Fig. 1d), suggested that the deprotonated silanol groups were negatively charged on the surface of nanoparticles. These DMSNs demonstrated characteristic IV type of the nitrogen adsorption–desorption isotherm (Fig. 1e, f) with a narrow pore size distribution (3.5 nm and 4.6 nm) (Fig. 1e, f, insert), indicating their mesoporous nanoshell structure. Taken together, monodisperse DMSNs with a similar mean particle size of 160 nm and different pore sizes of 3.5 nm and 4.6 nm, respectively, were successfully synthesized.

TGA was performed to determine the drug loading in the DMSNs (Additional file 1: Figure S2). The LC% of erianin was 53.08% and 46.66%, respectively, manifesting the high performance of the rotary evaporation technique for drug loading. The calculated values were consistent with erianin quantification by UPLC after extraction with absolute methanol (Additional file 1: Table S1).

The TEM images of E/DMSN_1_ and E/DMSN_2_ (Additional file 1: Figure S3), revealed that erianin coating on the DMSNs increased their aggregation. The average size values were determined by DLS as 225 nm and 207 nm, and zeta potential values as −25.2 mV and −26.5 mV for E/DMSN_1_ and E/DMSN_2_, respectively (Additional file 1: Table S2). Concerning the E/DMSNs, all of them exhibited the mean diameter slightly higher than the corresponding DMSNs, thus implying that the guest molecules partially depositing on the surface of the DMSNs increased their aggregation. On the other hand, erianin depositing on the surface of the DMSNs might change the diffusion coefficient leading to increase the hydrodynamic radius [25, 26]. This could be another reason for the increased mean diameter. Anyway, since the change of zeta potential values were small, it is hard to draw any conclusions about interactions between erianin and DMSNs.

Ntrogen adsorption–desorption isotherms results illustrated the SSA values of 274 m^2^ g^−1^ and 460 m^2^ g^−1^, pore volumes values of 0.289 cm^3^ g^−1^ and 0.523 cm^3^ g^−1^, and pore diameter values of 3.5 and 4.6 nm for DMSN_1_ and DMSN_2_, respectively (Additional file 1: Table S2). After drug loading, the SSA and pore volumes decreased to 24 m^2^ g^−1^ and 7 m^2^ g^−1^, and 0.057 m^2^ g^−1^ and 0.014 m^2^ g^−1^ for E/DMSN_1_ and E/DMSN_2_, respectively. Pore diameter also decreased to 2.6 nm and 3.2 nm for E/DMSN_1_ and E/DMSN_2_. This indicated the presence of erianin both inside the mesopores and in the silica porosity.

FTIR analysis was employed to study the interaction between erianin and DMSNs (Additional file 1: Figure S4). DMSNs exhibited a broad and large band at 3418 cm^−1^ due to intermolecular hydrogen bond interactions with surface DMSNs isolated silanol groups. Additionally, the sharp band at 1089 cm^−1^ was assigned to the asymmetric stretching vibrations (Si–O–Si). The spectrum of erianin showed the bands at 3539, 2939 and 1589 cm^−1^ characteristic for isolated –OH, C-H and phenyl bands. Coming to the E/DMSNs, the spectrum was demonstrated by intensity increase of the broad band at 3418 cm^−1^, due to erianin hydrogen (Additional file 1: Figure S5) bonding to surface DMSNs isolated silanol groups. Also, the characteristic band related to erianin was observed, confirming the presence and structural integrity of erianin molecules. Moreover, the intensity of the broad band at 3418 cm^−1^ of E/DMSN_1_ exhibited slightly higher than the corresponding E/DMSN_2_, indicating more hydrogen bonding interactions from the silica surface groups to the hydroxy of erianin. This result, in agreement with DLS data, TGA and UPLC measurements, suggested a higher amount of erianin deposited on the surface of DMSN_1_ than that of DMSN_2_.

The crystal properties of samples were studied by XRD analysis using wide-angle ranging from 5° to 60°. As shown in Additional file 1: Figure S6, the diffraction pattern of pure erianin was highly crystalline as revealed by the numerous peaks. These peaks were also present at a lower intensity in E/DMSNs, suggesting incomplete amorphous of erianin [27, 28]. These results were in good agreement with the aforementioned evidence, confirming the successful loading of erianin.

## In vitro release studies and intracellular uptake

**Fig. 2:**
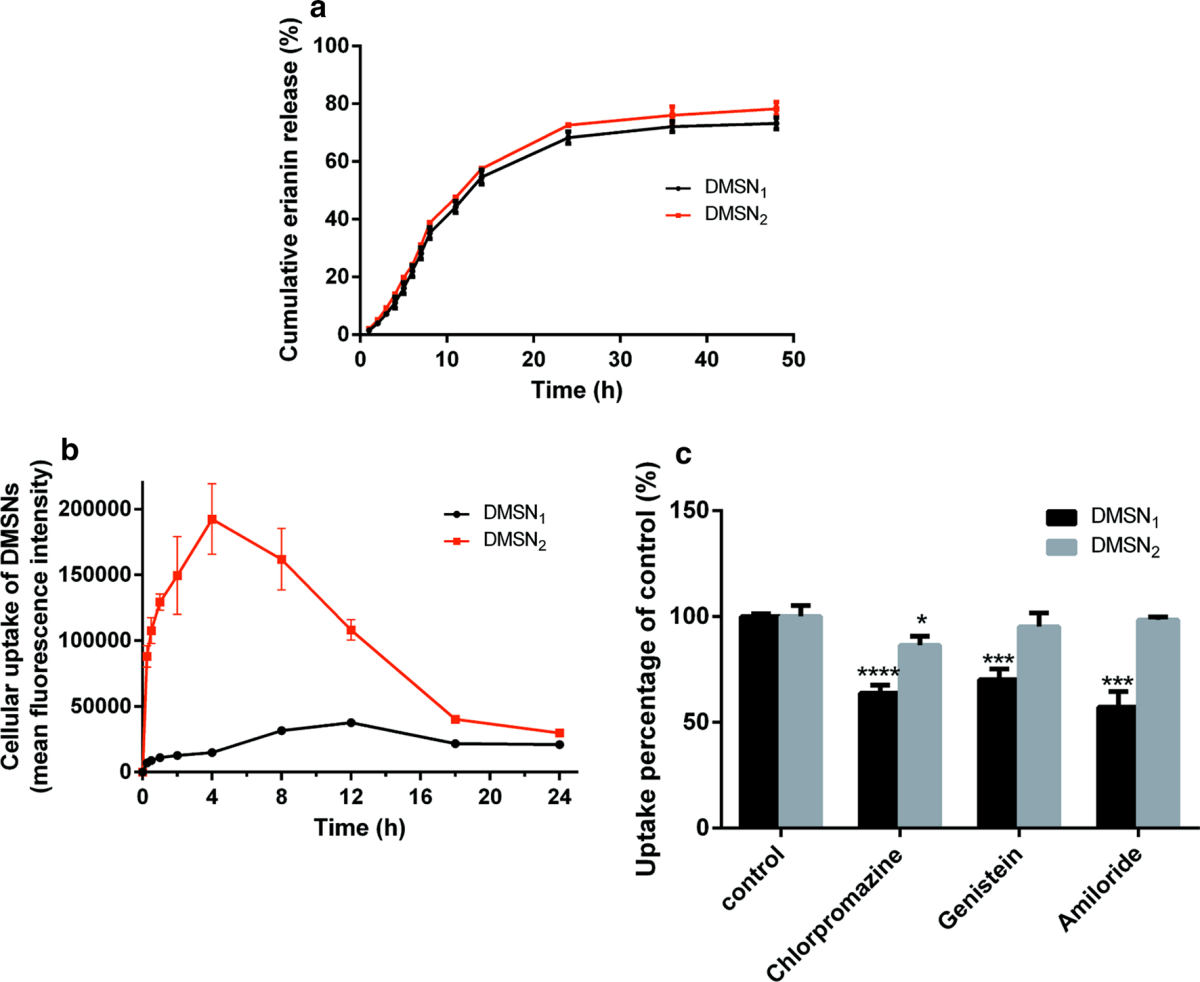
(**a**) Erianin release from E/DMSNs at pH: 7.4. (**b**) Flow cytometry analysis of cellular uptake of DMSNs. (**c**) Mechanisms of cellular uptake of DMSNs. Uptake percentage was normalized to particle uptake in the absence of inhibitors. The values are expressed as means ± SD (n = 3). *p < 0.05, ***p < 0.005, ****p < 0.001, significantly different compared with the control group

The cumulative release of erianin from DMSNs was investigated in PBS (pH = 7.4). As shown in Fig. 2a, the DMSNs released erianin in a controlled release manner. And the drug release rate of DMSN_2_ was slightly higher than of DMSN_1_. Initially, the erianin burst release reached 68% and 72% from DMSN_1_ and DMSN_2_ within 24 h, respectively, owing to the portion of erianin deposited on the outer surface of DMSNs. Later, erianin sustained release after 24 h and reached 73% and 76% from DMSN_1_ and DMSN_2_ within 48 h, respectively, indicating the Strong hydrogen bonding interactions between the silanol groups from mesopore channels and the erianin molecules.

To determine the uptake effect of two types of DMSNs of similar size and charge, varying only in the pore size, the flow cytometric analysis was carried out. As presented in Fig. 2b, the mean fluorescence intensity increased to a maximum at 4 h and 12 h for DMSN_2_ and DMSN_1_, respectively. The maximum mean fluorescence intensity (MFI) of DMSN_2_ was 3 times higher than of DMSN_1_, confirming that the DMSN_2_ were capable of faster uptake into the HaCaT cells than that of DMSN_1_. The larger pore size of DMSNs exhibited higher cellular uptake efficiency, which could be ascribed to the following reason [20]: the number of the DMSNs with larger pore size was greater than the smaller pore size of DMSNs at the same concentration, leading to more efficient cellular uptake of DMSNs. Moreover, the MFI decreased to a similar level at 24 h for both DMSNs, assuming that the uptake of both DMSNs achieved a dynamic balance between endocytosis and exocytosis [29]. Although the DMSNs were not degradable in cell media in this time window studied, oxidative and enzymatic degradation inside certain cells could also be a potential cause of the observed fluorescence decrease [30].

To further explore the specific mechanisms involved in cellular uptake of DMSNs, endocytic inhibitors were used to interfere with different uptake pathways. Specifically, chlorampromazine is a cationic amphiphilic drug that inhibits specifically the clathrin-mediated pathway [31]. Genistein blocks caveolae-mediated endocytosis by inhibiting the Src tyrosine kinase phosphorylation of caveolin-1 and preventing vesicle fusion [32]. Amiloride is an inhibitor of Na^+^/H^+^ exchanger involvement of macropinocytosis [33]. These inhibitors were utilized under nontoxic concentrations (Additional file 1: Figure S7). As shown in Fig. 2c, the cellular uptake of DMSN_2_ was prohibited to a smaller degree in the presence of chlorampromazine. And the cellular uptake of DMSN_1_ was inhibited to a larger degree in the presence of chlorampromazine, genistein, and amiloride. As a result, the cellular uptake of DMSN_2_ was via clathrin-mediated endocytosis pathway and the DMSN_1_ could get into cells in a different strategy. The cellular uptake efficiency is not only depends on the number of endocytosis pathways, but also their transport capacity for each nanoparticle [30]. On the other hand, structure–function relationship can not be neglected. Methyl Salicylate and methyl parahydroxybenzoats had similar structure but showed distinct pharmacological effects because of different molecular conformation. In our study, for DMSN_1_, the silanol groups from channels and surface are more dense, which may induce stronger interactions with the membrane receptors, and that might explain why DMSN_1_ exhibited more endocytosis pathways. However, the clathrin-mediated endocytosis pathway may suffer from the influence of pH and the lack of lysosomal [34]. The DMSN_1_ with several cellular uptake pathways therefore could be a prospective drug delivery system in a diversity of physiological environment.

## E/DMSNs inhibited proliferation and induced apoptosis in HaCaT cells

**Fig. 3:**
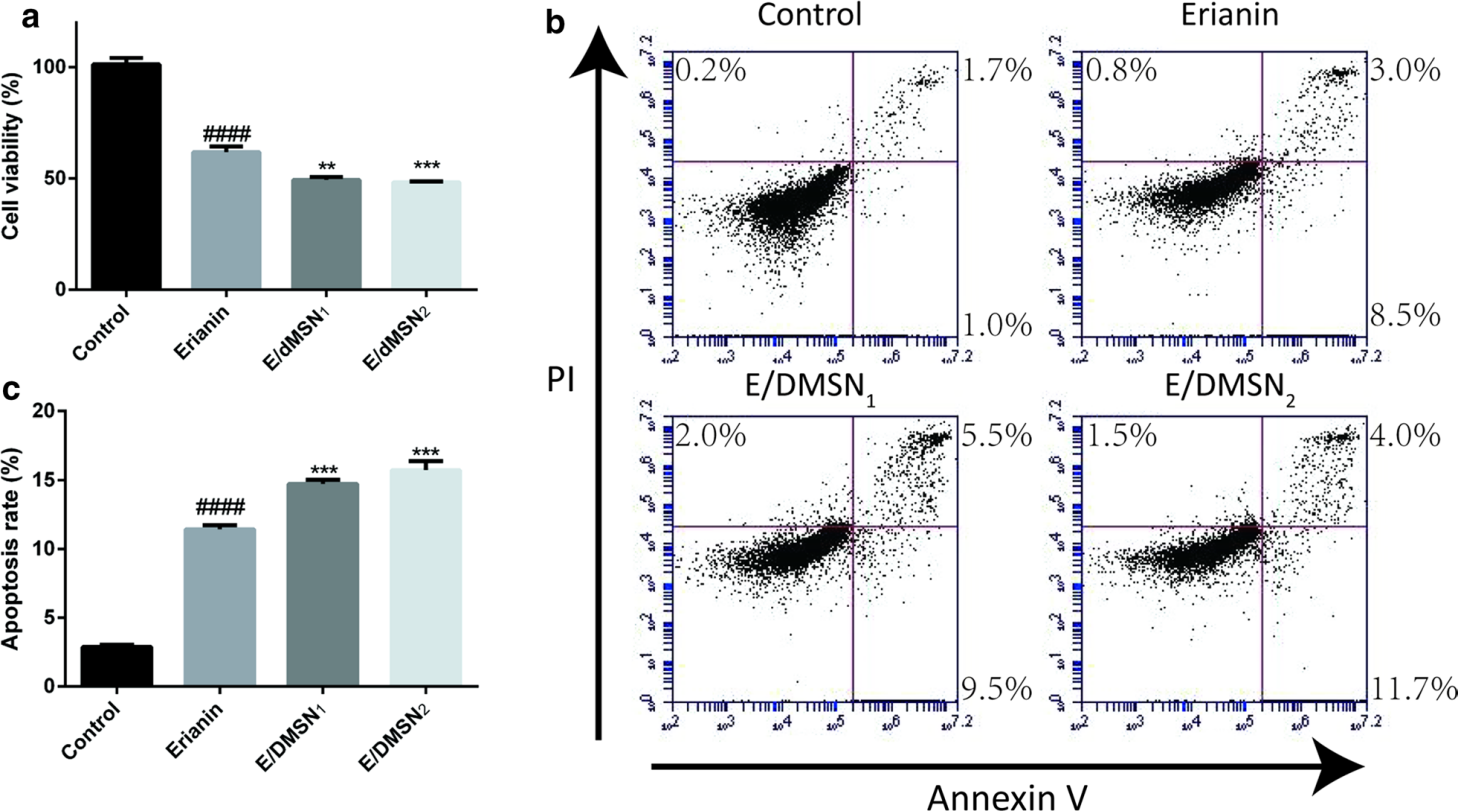
Effect of erianin and E/DMSNs on the viability and apoptosis in HaCaT cells **a** Cell viability of erianin and E/DMSNs in HaCaT cells for 24 h. **b** Flow cytometry analysis of early and apoptotic HaCaT cells after treatment with erianin and E/DMSNs for 24 h. **c** Quantitative analysis of apoptosis rate in (**b**). Values are represented as means ± SD (n = 3). **p < 0.01, ***p < 0.005, significantly different compared with the erianin group. ####*p* < 0.001, significantly different compared with the control group

In order to evaluate the anti-proliferative effect of E/DMSNs in HaCaT cells, we used MTT assays to study cell viability. Firstly, the viability of HaCaT cells after 24 h of incubation with DMSNs did not exhibit notable cytotoxicity even when the concentration was as high as 0.1 mg/mL (Additional file 1: Figure S8), indicating their excellent biocompatibility. As shown in Fig. 3a, the cell viability of erianin, E/DMSN_1_, and E/DMSN_2_ was 61.8%, 49.3%, and 48.3%, respectively, indicating an enhancement anti-proliferative effect of E/DMSNs.

Then we further investigated the pro-apoptotic effect of E/DMSNs in HaCaT cells through Annexin V-FITC/PI staining with flow cytometry. Annexin V, a calcium ion-dependent phospholipid-binding protein, has a high affinity for membrane phospholipid phosphatidylserine, which migrates through the cell membrane from the inner to the outer of the lipid bilayer in the early stage of cell apoptosis. PI, a nucleic acid dye, can only across cell membrane of the late stage of cell apoptosis and dead cell. Hence, Annexin V-FITC/PI staining could detect the early apoptosis and the late apoptosis/dead cells. As presented in Fig. 3b, in the flow cytometry scatter diagram, the lower left, lower right, and upper right quadrants indicated living, early apoptotic and late apoptotic cells regions, respectively. In the erianin group, the percentage of the early stage of cell apoptosis and the late stage of cell apoptosis reached 8.5% and 3.0%. In the E/DMSN_1_ group, the percentage of early apoptotic cell population and late apoptotic cell population increased to 9.5% and 5.5%. Similarly, E/DMSN_2_ incubation increased the early and late apoptotic percentage to 11.7% and 4.0%. Taken together, as shown in Fig. 3c, all E/DMSNs showed superior pro-apoptotic effect to erianin, and E/DMSN_2_ with larger pore size demonstrated the enhanced pro-apoptotic effect, which was consistent with the anti-proliferative results. It is conceivable that the increased anti-proliferative and pro-apoptotic effect of E/DMSNs with an increase in pore size might be attributable to the pore size-dependent cellular uptake of DMSNs, which had been confirmed by quantitative measurement of the cellular uptake as mentioned above.

## E/DMSNs induced apoptosis through the mitochondrial signaling pathway in HaCaT cells

**Fig. 4:**
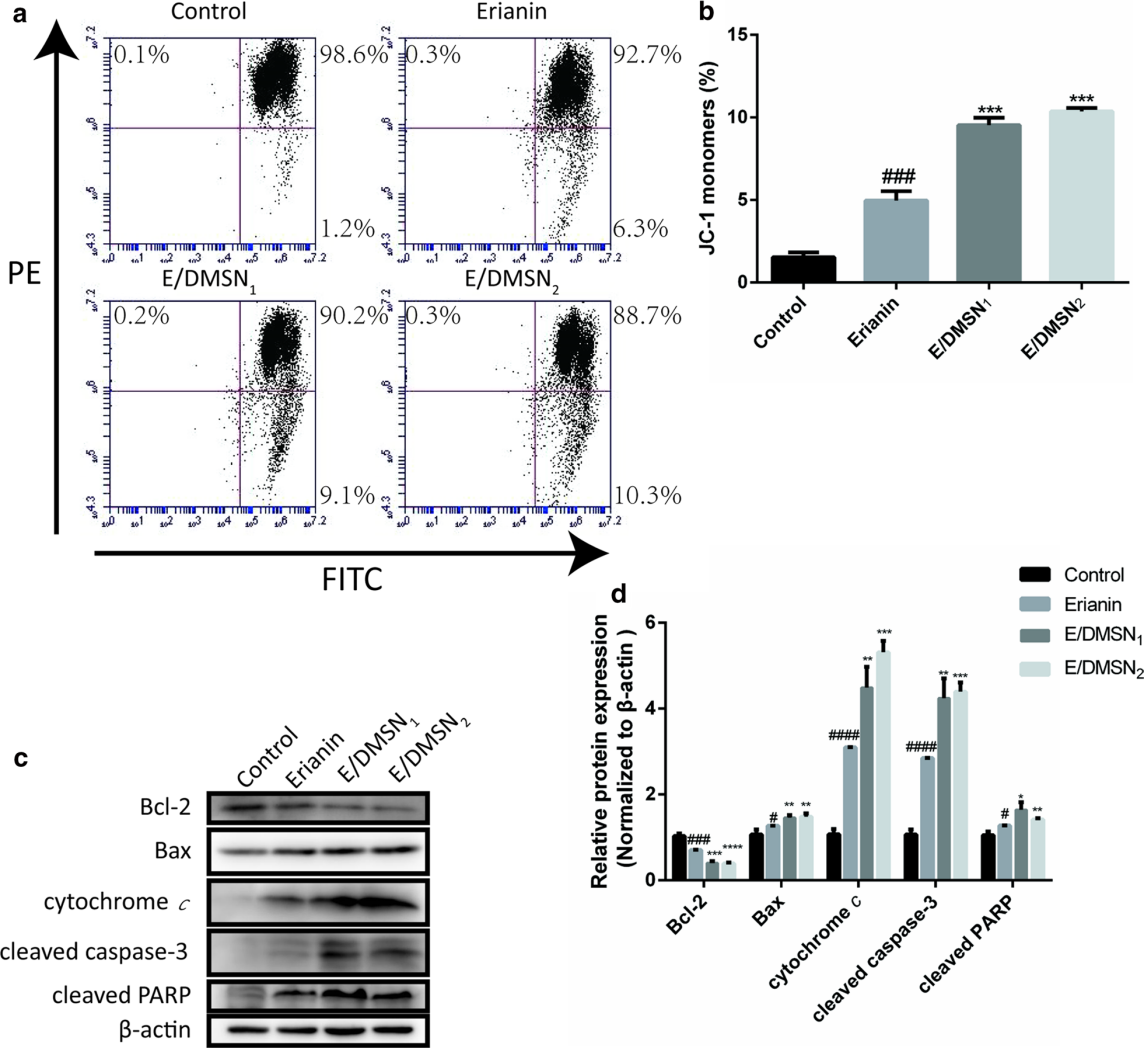
Effect of erianin and E/DMSNs on mitochondrial membrane potential and mitochondrial signaling pathway. **a** Flow cytometry analysis of mitochondrial membrane potential in HaCaT cells after treatment with erianin and E/DMSNs for 24 h. **b** Quantitative analysis of the percentage of JC-1 monomers rate in (**a**). **c** The expressions of Bcl-2, Bax, cytochrome *c*, cleaved caspase-3, and cleaved PARP proteins after treatment with erianin and E/DMSNs for 24 h. **d** Quantitation of Bcl-2, Bax, cytochrome *c*, cleaved caspase-3, and cleaved PARP proteins normalized to β-actin in (**c**) by using Image J software. Values are represented as means ± SD (n = 3). *p < 0.05, **p < 0.01, ***p < 0.005, ****p < 0.001, significantly different compared with the erianin group. #*p* < 0.05, ###*p* < 0.005, ####*p* < 0.001, significantly different compared with the control group

We further explored the effects of E/DMSNs on the mitochondrial signaling pathway. The BCL-2 family of proteins, consisting of anti-apoptotic proteins and pro-apoptotic proteins (Bcl-2 and Bax, respectively), constitutes a critical cellular checkpoint in mitochondrial-mediated apoptosis [35]. Downregulation of Bcl-2 can promote the translocation of Bax to mitochondria, which will from pores capable of releasing cytochrome *c* from mitochondria into the cytoplasm and reduce mitochondrial membrane potential. Cytochrome *c* subsequently activates caspase-3 and PARP, which ultimately induces cell apoptosis [36]. Fluorescent mitochondrial probe JC-1 was employed to measure mitochondrial membrane potential through flow cytometry. JC-1 forms red-fluorescent aggregates at low membrane potential (living cells) and converts to green-fluorescent monomers at high membrane potential (apoptotic cells). The flow cytometry scatter diagram showed that cells treated with E/DMSNs showed a heavy shift of cell population from the upper right quadrant towards the lower right quadrant (Fig. 4a), and the percentage of JC-1 monomers (E/DMSN_1_ and E/DMSN_2_) was significantly increased to 9.5% and 10.3% of the erianin group (4.9%) level, respectively (Fig. 4b), indicating an enhancement of mitochondrial depolarization effect of E/DMSNs. Next, we investigated the expression of apoptosis-related proteins by western blotting. As shown in Fig. 4c, d, an obvious increase in the activation of Bax, cytochrome *c*, cleavage caspase-3 and of cleaved PARP, and the expression of Bcl-2 was reduced. In summary, these results indicated that E/DMSNs provoked cell apoptosis via the mitochondrial signaling pathway.

## E/DMSNs induced apoptosis through regulation of endoplasmic reticulum stress in HaCaT cells

**Fig. 5:**
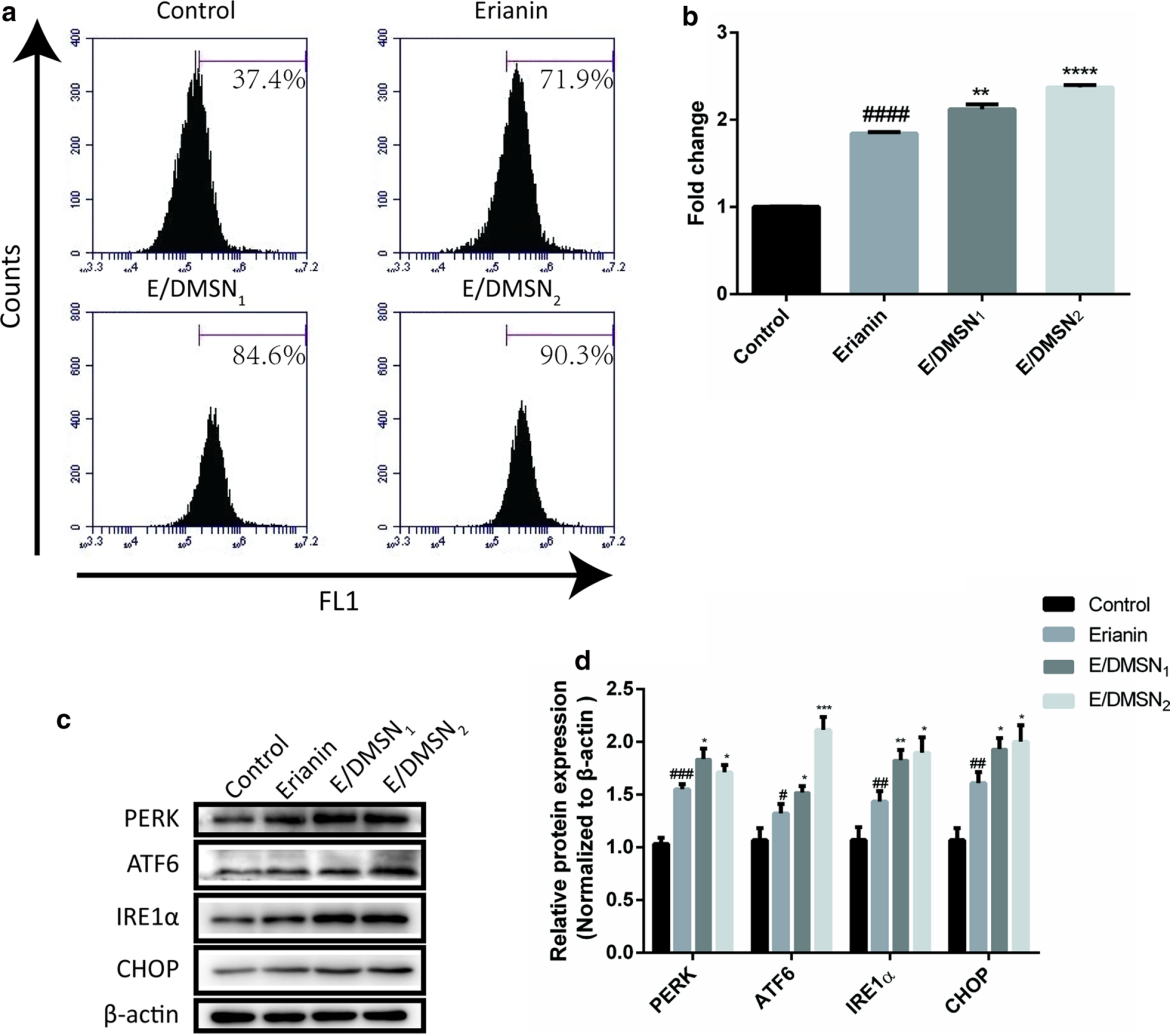
Effect of erianin and E/DMSNs on cytosolic calcium levels and ERS signaling pathway. **a** Flow cytometry analysis of cytosolic calcium levels in HaCaT cells after treatment with erianin and E/DMSNs for 24 h. **b** Relative MFI of control in (**a**) analyzed by flow cytometry. **c** The expressions of PERK, ATF6, IRE1α, and CHOP proteins after treatment with erianin and E/DMSNs for 24 h. **d** Quantitation of PERK, ATF6, IRE1α, and CHOP proteins normalized to β-actin in (**c**) by using Image J software. Values are represented as means ± SD (n = 3). *p < 0.05, **p < 0.01, ***p < 0.005, ****p < 0.001, significantly different compared with the erianin group. ##*p* < 0.01, ###*p* < 0.005, ####*p* < 0.001, significantly different compared with the control group

Accumulating evidence in recent years demonstrates that in addition to mitochondria, the endoplasmic reticulum plays a significant role in the apoptotic control point [37]. Much physiological stimulation may cause ERS leading the accumulation of unfolded proteins and excess release of calcium ion into the cytosol from ER. To maintain homeostasis of protein synthesis and calcium, ERS activates a cytoprotective response termed unfolded protein response (UPR). When homeostasis fails, the UPR can act as an apoptotic executor that scavenges cells. The UPR mediates ERS requiring the activation of three ER transmembrane signal transducers: PERK, ATF6, and IRE1 [38]. The activation of PERK pathway and ATF6 pathway upregulates CHOP, a crucial pro-apoptotic transcription factor during ER-mediated apoptosis, which in turn downregulates the anti-apoptotic protein Bcl-2 [39]. In addition, continued activation of IRE1 can directly interact with pro-apoptotic protein Bax and inactivates Bcl-2 protein through c-Jun N-terminal kinase (JNK) pathway [40, 41]. Moreover, excess release of calcium ion from the ER is recruited by mitochondria, causing the decrease of mitochondrial membrane potential and the leakage of cytochrome *c *[42]. Taken together, the cross-talk between the ER and mitochondria leads to cell death. Consequently, we measured the cytosolic calcium levels in HaCaT cells stained with Fluo-4 AM and investigated the expression of ERS-associated proteins by western blotting. As shown in Fig. 5 a, b, the cells treated with E/DMSNs showed a shift of the peak of cellular fluorescence compared to the erianin group. And the fold change of the cytosolic calcium levels increased to 1.8, 2.1, and 2.3 for erianin, E/DMSN_1_, and E/DMSN_2_ group, respectively, compared to the control group. As shown in Fig. 5 c, d, the immunoblot analysis in HaCaT cells revealed that the cells treated with E/DMSNs exhibited the upregulation of PERK, ATF6, IRE1α, and CHOP levels compared to control group as well as erianin group. The results altogether demonstrated that E/DMSNs induced cell apoptosis through regulation of endoplasmic reticulum stress.

## In vitro skin permeation studies

**Fig. 6:**
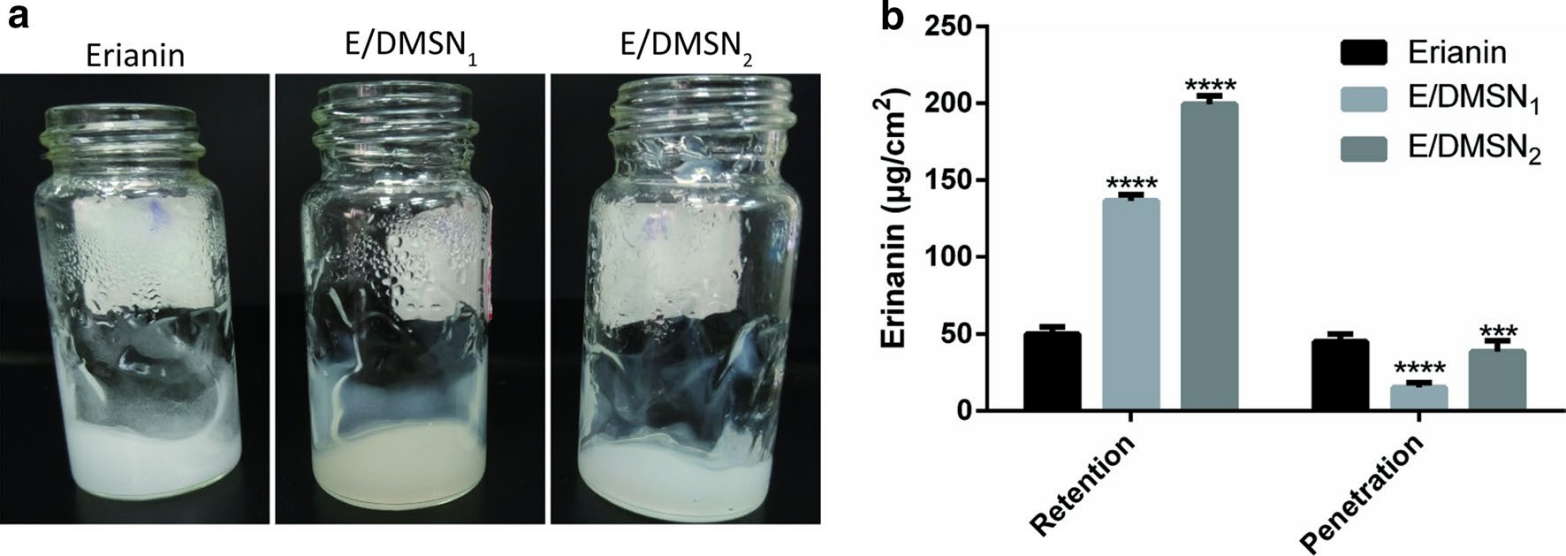
**a** Images of erianin gel, E/DMSN_1_ gel, and E/DMSN_2_ gel. **b** Amounts of erianin accumulated in the skin and permeated through the skin after 24 h application. The receptor chamber was PBS/ethanol (80/20, v/v) and was continuously stirred at 37 °C. Values are represented as means ± SD (n = 3). ***p < 0.005, ****p < 0.001, significantly different compared with the erianin group

Carbopol gel was used as the matrix of erianin and E/DMSNs to achieve homogeneous and proper viscosity of erianin loaded formulations for suitable to the transdermal application. The addition of the gel assured the consistency among the formulations and extended the interaction time between the drug and the skin. The in vitro percutaneous permeability of erianin gel, E/DMSN_1_ gel, and E/DMSN_2_ gel through porcine ear skin was investigated using Franz diffusion cells. A mixture of PBS/ethanol (80/20, v/v) was chosen as receiving phase to ensure the complete dissolution of erianin. Furthermore, as antimicrobial component, ethanol was added to the receptor fluid for the purpose of preventing skin deterioration during experiment [18]. As shown in Fig. 6b, much higher drug retentions in the skin were obtained for both E/DMSNs gel, compared to the erianin gel group. In addition, the E/DMSN_2_ gel showed higher accumulation of drug than that of E/DMSN_1_ gel, which might be attributable to the following reason: the surface area of the DMSNs with larger pore size was larger than the smaller pore size of DMSNs, facilitating the longer retention and release of loaded drug in the skin. Moreover, lower drug penetrations from the skin were obtained for both E/DMSNs gel, compared to the erianin gel group, indicating the better topical application performance and less systemic toxicity.

## Conclusions

In this work, two pore sizes of DMSNs were investigated for in vitro drug release, cellular uptake behavior, endocytosis mechanism, and ex vivo permeation study on porcine ear skin. In addition, the biological roles of these DMSNs were evaluated in HaCaT cells. Compared to free erianin, E/DMSNs showed pore-size-dependent, higher anti-proliferative and pro-apoptotic effects against HaCaT cells through mitochondrial and ERS signaling pathway, and exhibited higher drug retention and less drug penetration in the skin. The potential mechanism could be due to the difference in cellular uptake rate and surface area, resulting from the difference of pore size. We have to mention that DMSN_1_ as a drug carrier, with smaller pore size, might be proficient in the complicated physiological environment, owing to multiple cellular uptake strategies. These results illustrated that DMSNs could be very promising drug delivery systems for therapy of keratinocyte-related diseases such as psoriasis.

## Supplementary information

**Additional file 1: Figure S1.** Tem images of (**a**) DMSN1 and (**b**) DMSN2.** Figure S2.** TGA of DMSNs and E/DMSNs. Weight losses were determined in nitrogen flow up to 1000 °C.** Figure S3.** TEM images of (**a**) E/DMSN1 and (**b**) E/DMSN2.** Table S1.** Data from TGA (percentage weight loss) and from UPLC analysis (LC%).** Table S2.** Textural properties of the silica-based samples.** Figure S4.** FITR spectra of DMSN1, erian, and E/DMSN1 in (**a**) and of DMSN2, erian, and E/DMSN2 in (**b**).** Figure S5.** The chemical structure of erianin.** Figure S6.** wide angle XRD analysis of all samples.** Figure S7.** Cytotoxicity evaluation of three inhibitors in HaCaT cells for 24 h.** Figure S8. **Cytotoxicity evaluation of DMSNs in HaCaT cells for 24 h.

## Data Availability

All data generated or analyzed during this study are included in this published article and its additional file.

## Abbreviations

E/DMSNs: Erianin loaded dendritic mesoporous silica nanospheres
HaCaT: Human immortalized keratinocyte
ERS: Endoplasmic reticulum stress
JNK: c-Jun N-terminal kinase
CTAC: Cetyltrimethylammonium chloride
TEA: Triethanolamine
TEOS: Tetraethyl orthosilicate
APTES: 3-Aminopropyltriethoxysilane
FITC: Fluorescein isothiocyanate
DMEM: Dulbecco modified Eagle medium
FBS: Fetal bovine serum
MTT: 3-(4,5-dimethylthiazol-2-yl)-2,5-dipheny-ltetrazolium bromide
DMSO: Dimethyl sulfoxide
PI: Propidium iodide
PARP: Poly (ADP-ribose) Polymerase
ATF6: Activating transcription factor 6
IRE1α: Inositol-requiring enzyme 1α
PERK: Protein kinase RNA-like ER kinase
CHOP: c/EBP Homologous protein
LC%: Loading capacity
TEM: Transmission electron microscopy
DLS: Dynamic light scattering
SSA: Specific surface areas
TGA: Thermogravimetric analysis
FTIR: Fourier transform infrared
XRD: Powder X-ray diffractograms
PBS: Phosphate buffered solution
UPLC: Ultra-high performance liquid chromatography
RIPA: Radio-immunoprecipitation assay
PVDF: Poly (vinylidene fluoride)
TBST: Tris-buffered saline and Tween 20 buffer
MFI: Mean fluorescence intensity
UPR: Unfolded protein response

## References

[1] Boehncke WH, Schon MP (2015). Psoriasis. Lancet.

[2] Andon FT, Fadeel B (2013). Programmed cell death: molecular mechanisms and implications for safety assessment of nanomaterials. Acc Chem Res.

[3] Delbridge AR, Grabow S, Strasser A, Vaux DL (2016). Thirty years of BCL-2: translating cell death discoveries into novel cancer therapies. Nat Rev Cancer.

[4] Flusberg DA, Sorger PK (2015). Surviving apoptosis: life-death signaling in single cells. Trends Cell Biol.

[5] Walter P, Ron D (2011). The unfolded protein response: from stress pathway to homeostatic regulation. Science.

[6] Zhong SY, Dong YY, Liu DX, Xu DH, Xiao SX, Chen HL, Kong MG (2016). Surface air plasma-induced cell death and cytokine release of human keratinocytes in the context of psoriasis. Br J Dermatol.

[7] Datta Mitra A, Raychaudhuri SP, Abria CJ, Mitra A, Wright R, Ray R, Kundu-Raychaudhuri S (2013). 1alpha,25-Dihydroxyvitamin-D3-3-bromoacetate regulates AKT/mTOR signaling cascades: a therapeutic agent for psoriasis. J Invest Dermatol.

[8] Gong YQ, Fan Y, Wu DZ, Yang H, Hu ZB, Wang ZT (2004). In vivo and in vitro evaluation of erianin, a novel anti-angiogenic agent. Eur J Cancer.

[9] Wang H, Zhang T, Sun W, Wang Z, Zuo D, Zhou Z, Li S, Xu J, Yin F, Hua Y, Cai Z (2016). Erianin induces G2/M-phase arrest, apoptosis, and autophagy via the ROS/JNK signaling pathway in human osteosarcoma cells in vitro and in vivo. Cell Death Dis.

[10] Mo C, Shetti D, Wei K (2019). Erianin inhibits proliferation and induces apoptosis of HaCaT cells via ROS-mediated JNK/c-Jun and AKT/mTOR signaling pathways. Molecules.

[11] Pradhan M, Singh D, Singh MR (2013). Novel colloidal carriers for psoriasis: current issues, mechanistic insight and novel delivery approaches. J Control Release.

[12] Mezei M, Gulasekharam V (1980). Liposomes–a selective drug delivery system for the topical route of administration Lotion dosage form. Life Sci.

[13] Kim JY, Ahn J, Kim J, Choi M, Jeon H, Choe K, Lee DY, Kim P, Jon S (2018). Nanoparticle-assisted transcutaneous delivery of a signal transducer and activator of transcription 3-inhibiting peptide *Ameliorates psoriasis*-like skin inflammation. ACS Nano.

[14] Zaric M, Lyubomska O, Touzelet O, Poux C, Al-Zahrani S, Fay F, Wallace L, Terhorst D, Malissen B, Henri S (2013). Skin dendritic cell targeting via microneedle arrays laden with antigen-encapsulated poly-d, l-lactide-co-glycolide nanoparticles induces efficient antitumor and antiviral immune responses. ACS Nano.

[15] Mei L, Zhang Z, Zhao L, Huang L, Yang XL, Tang J, Feng SS (2013). Pharmaceutical nanotechnology for oral delivery of anticancer drugs. Adv Drug Deliv Rev.

[16] Berlier G, Gastaldi L, Sapino S, Miletto I, Bottinelli E, Chirio D, Ugazio E (2013). MCM-41 as a useful vector for rutin topical formulations: synthesis, characterization and testing. Int J Pharm.

[17] Gupta R, Rai B (2018). In-silico design of nanoparticles for transdermal drug delivery application. Nanoscale.

[18] Ugazio E, Gastaldi L, Brunella V, Scalarone D, Jadhav SA, Oliaro-Bosso S, Zonari D, Berlier G, Miletto I, Sapino S (2016). Thermoresponsive mesoporous silica nanoparticles as a carrier for skin delivery of quercetin. Int J Pharm.

[19] Jia L, Shen J, Li Z, Zhang D, Zhang Q, Liu G, Zheng D, Tian X (2013). In vitro and in vivo evaluation of paclitaxel-loaded mesoporous silica nanoparticles with three pore sizes. Int J Pharm.

[20] Gao Y, Chen Y, Ji X, He X, Yin Q, Zhang Z, Shi J, Li Y (2011). Controlled intracellular release of doxorubicin in multidrug-resistant cancer cells by tuning the shell-pore sizes of mesoporous silica nanoparticles. ACS Nano.

[21] Shen D, Yang J, Li X, Zhou L, Zhang R, Li W, Chen L, Wang R, Zhang F, Zhao D (2014). Biphase stratification approach to three-dimensional dendritic biodegradable mesoporous silica nanospheres. Nano Lett.

[22] Summerlin N, Qu Z, Pujara N, Sheng Y, Jambhrunkar S, McGuckin M, Popat A (2016). Colloidal mesoporous silica nanoparticles enhance the biological activity of resveratrol. Colloids Surf B Biointerfaces.

[23] Morelli C, Maris P, Sisci D, Perrotta E, Brunelli E, Perrotta I, Panno ML, Tagarelli A, Versace C, Casula MF (2011). PEG-templated mesoporous silica nanoparticles exclusively target cancer cells. Nanoscale.

[24] Sun L, Liu Z, Wang L, Cun D, Tong HHY, Yan R, Chen X, Wang R, Zheng Y (2017). Enhanced topical penetration, system exposure and anti-psoriasis activity of two particle-sized, curcumin-loaded PLGA nanoparticles in hydrogel. J Control Release.

[25] Kozer N, Kuttner YY, Haran G, Schreiber G (2007). Protein-protein association in polymer solutions: from dilute to semidilute to concentrated. Biophys J.

[26] Mallol R, Rodriguez MA, Heras M, Vinaixa M, Plana N, Masana L, Morris GA, Correig X (2012). Particle size measurement of lipoprotein fractions using diffusion-ordered NMR spectroscopy. Anal Bioanal Chem.

[27] Zhang Y, Zhi Z, Jiang T, Zhang J, Wang Z, Wang S (2010). Spherical mesoporous silica nanoparticles for loading and release of the poorly water-soluble drug telmisartan. J Control Release.

[28] Zhu W, Wan L, Zhang C, Gao Y, Zheng X, Jiang T, Wang S (2014). Exploitation of 3D face-centered cubic mesoporous silica as a carrier for a poorly water soluble drug: influence of pore size on release rate. Mater Sci Eng C Mater Biol Appl.

[29] Bartczak D, Nitti S, Millar TM, Kanaras AG (2012). Exocytosis of peptide functionalized gold nanoparticles in endothelial cells. Nanoscale.

[30] Agarwal R, Singh V, Jurney P, Shi L, Sreenivasan SV, Roy K (2013). Mammalian cells preferentially internalize hydrogel nanodiscs over nanorods and use shape-specific uptake mechanisms. Proc Natl Acad Sci USA.

[31] Bannunah AM, Vllasaliu D, Lord J, Stolnik S (2014). Mechanisms of nanoparticle internalization and transport across an intestinal epithelial cell model: effect of size and surface charge. Mol Pharm.

[32] Schulz WL, Haj AK, Schiff LA (2012). Reovirus uses multiple endocytic pathways for cell entry. J Virol.

[33] Bhowmick T, Berk E, Cui X, Muzykantov VR, Muro S (2012). Effect of flow on endothelial endocytosis of nanocarriers targeted to ICAM-1. J Control Release.

[34] Conner SD, Schmid SL (2003). Regulated portals of entry into the cell. Nature.

[35] Moldoveanu T, Follis AV, Kriwacki RW, Green DR (2014). Many players in BCL-2 family affairs. Trends Biochem Sci.

[36] Czabotar PE, Lessene G, Strasser A, Adams JM (2014). Control of apoptosis by the BCL-2 protein family: implications for physiology and therapy. Nat Rev Mol Cell Biol.

[37] Han J, Back SH, Hur J, Lin YH, Gildersleeve R, Shan J, Yuan CL, Krokowski D, Wang S, Hatzoglou M (2013). ER-stress-induced transcriptional regulation increases protein synthesis leading to cell death. Nat Cell Biol.

[38] Hetz C, Papa FR (2018). The Unfolded protein response and cell fate control. Mol Cell.

[39] Kang MK, Park SH, Kim YH, Lee EJ, Antika LD, Kim DY, Choi YJ, Kang YH (2017). Chrysin ameliorates podocyte injury and slit diaphragm protein loss via inhibition of the PERK-eIF2alpha-ATF-CHOP pathway in diabetic mice. Acta Pharmacol Sin.

[40] Hetz C, Bernasconi P, Fisher J, Lee AH, Bassik MC, Antonsson B, Brandt GS, Iwakoshi NN, Schinzel A, Glimcher LH, Korsmeyer SJ (2006). Proapoptotic BAX and BAK modulate the unfolded protein response by a direct interaction with IRE1alpha. Science.

[41] Fan M, Goodwin M, Vu T, Brantley-Finley C, Gaarde WA, Chambers TC (2000). Vinblastine-induced phosphorylation of Bcl-2 and Bcl-XL is mediated by JNK and occurs in parallel with inactivation of the Raf-1/MEK/ERK cascade. J Biol Chem.

[42] Cao SS, Kaufman RJ (2014). Endoplasmic reticulum stress and oxidative stress in cell fate decision and human disease. Antioxid Redox Signal.

