# Supplementary material for: Development of erianin-loaded dendritic mesoporous silica nanospheres with pro-apoptotic effects and enhanced topical delivery
Source: J Nanobiotechnology. 2020 Mar 30;18:55. doi: 10.1186/s12951-020-00608-3 (PMC7104482; doi:10.1186/s12951-020-00608-3)
Supplement: Supplementary file 1 — Additional file 1: Figure S1. Tem images of (a) DMSN1 and (b) DMSN2. Figure S2. TGA of DMSNs and E/DMSNs. Weight losses were determined in nitrogen flow up to 1000 °C. Figure S3. TEM images of (a) E/DMSN1 and (b) E/DMSN2. Table S1. Data from TGA (percentage weight loss) and from UPLC analysis (LC%). Table S2. Textural properties of the silica-based samples. Figure S4. FITR spectra of DMSN1, erian, and E/DMSN1 in (a) and of DMSN2, erian, and E/DMSN2 in (b). Figure S5. The chemical structure of erianin. Figure S6. wide angle XRD analysis of all samples. Figure S7. Cytotoxicity evaluation of three inhibitors in HaCaT cells for 24 h. Figure S8. Cytotoxicity evaluation of DMSNs in HaCaT cells for 24 h. [file 12951_2020_608_MOESM1_ESM.docx]

**
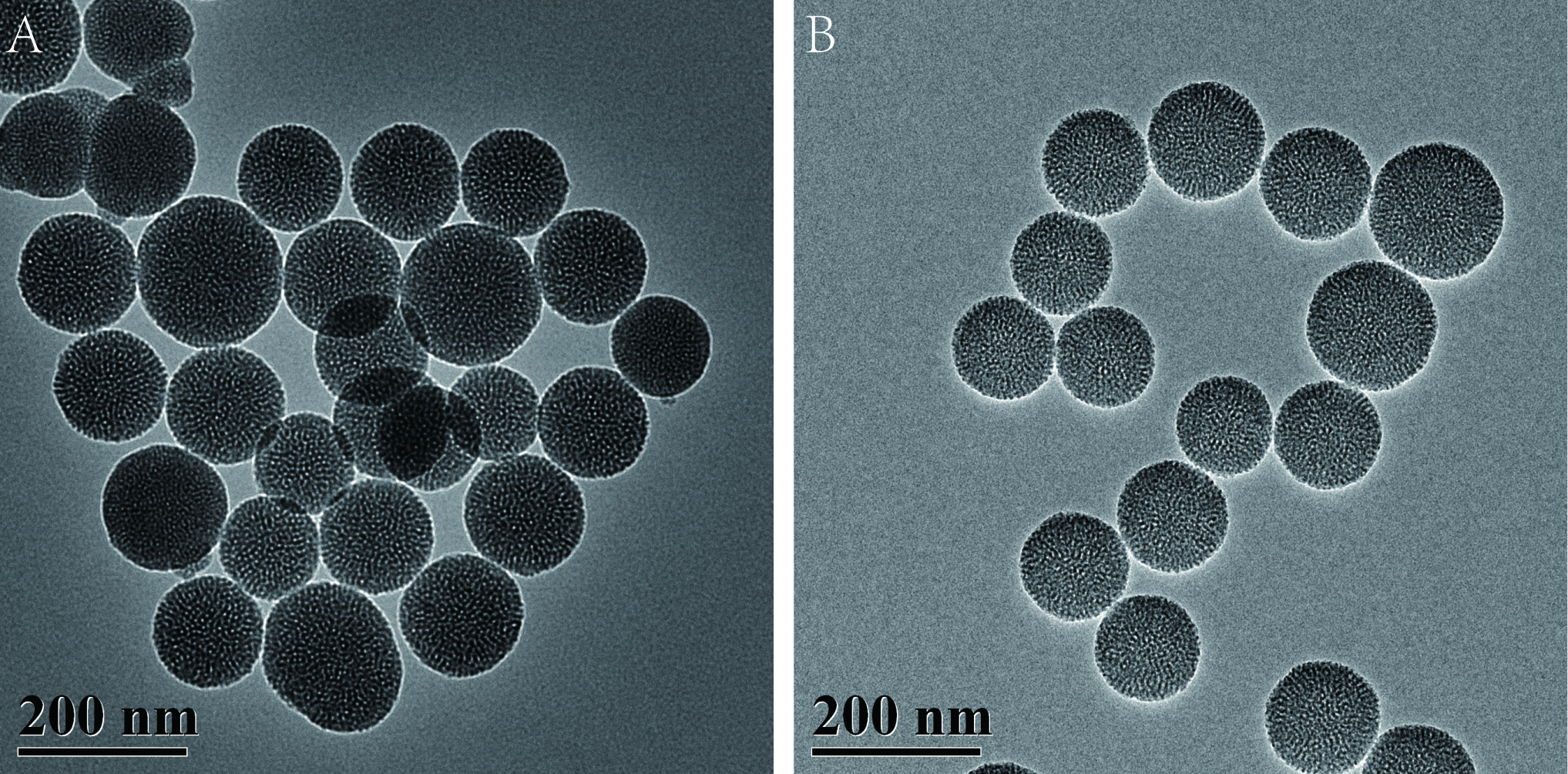
**

**Fig.S1** TEM images of (A) DMSN_1_ and (B) DMSN_2_.

**
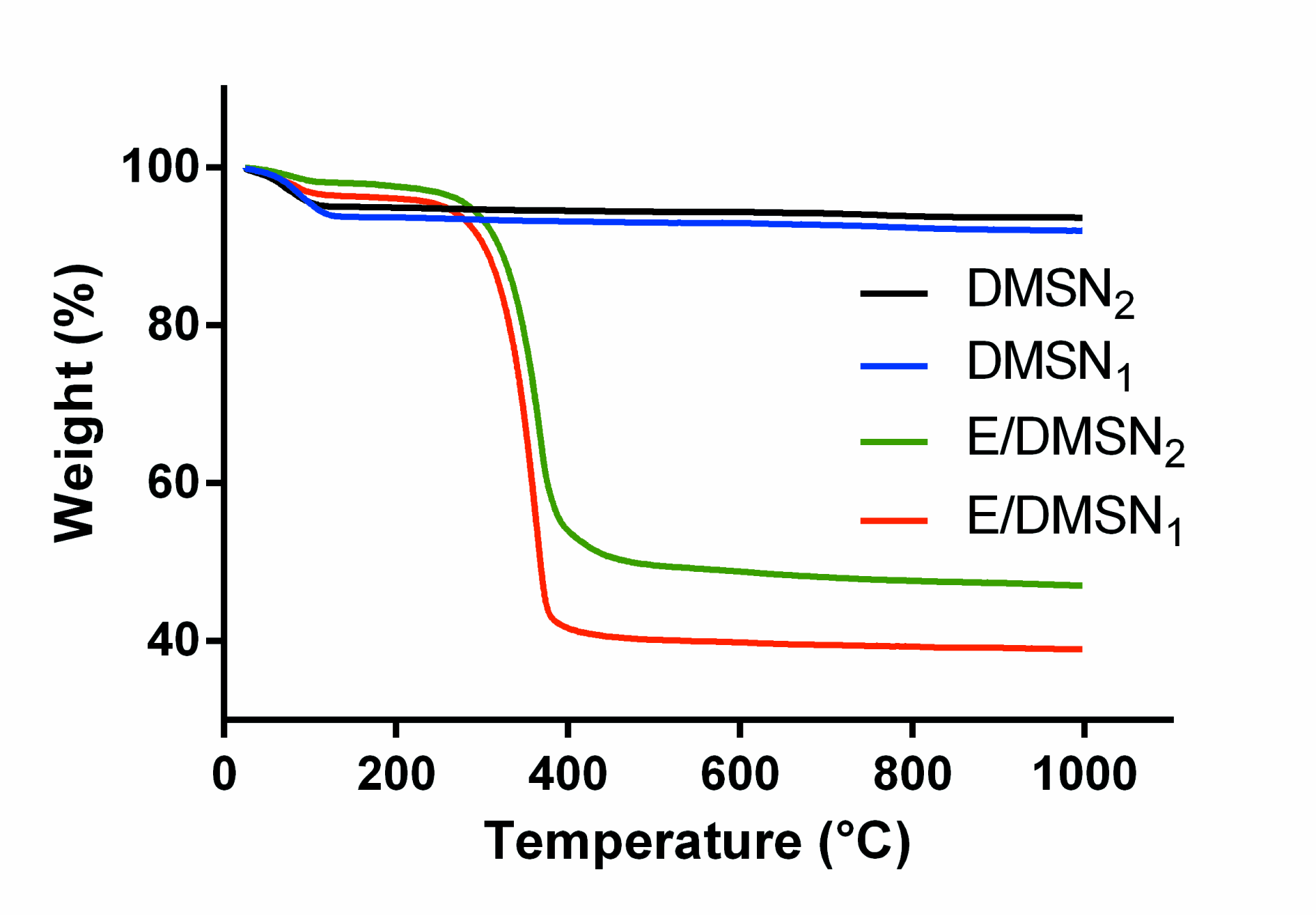
**

**Fig. S2** TGA of DMSNs and E/DMSNs. Weight losses were determined in nitrogen flow up to 1000 °C.


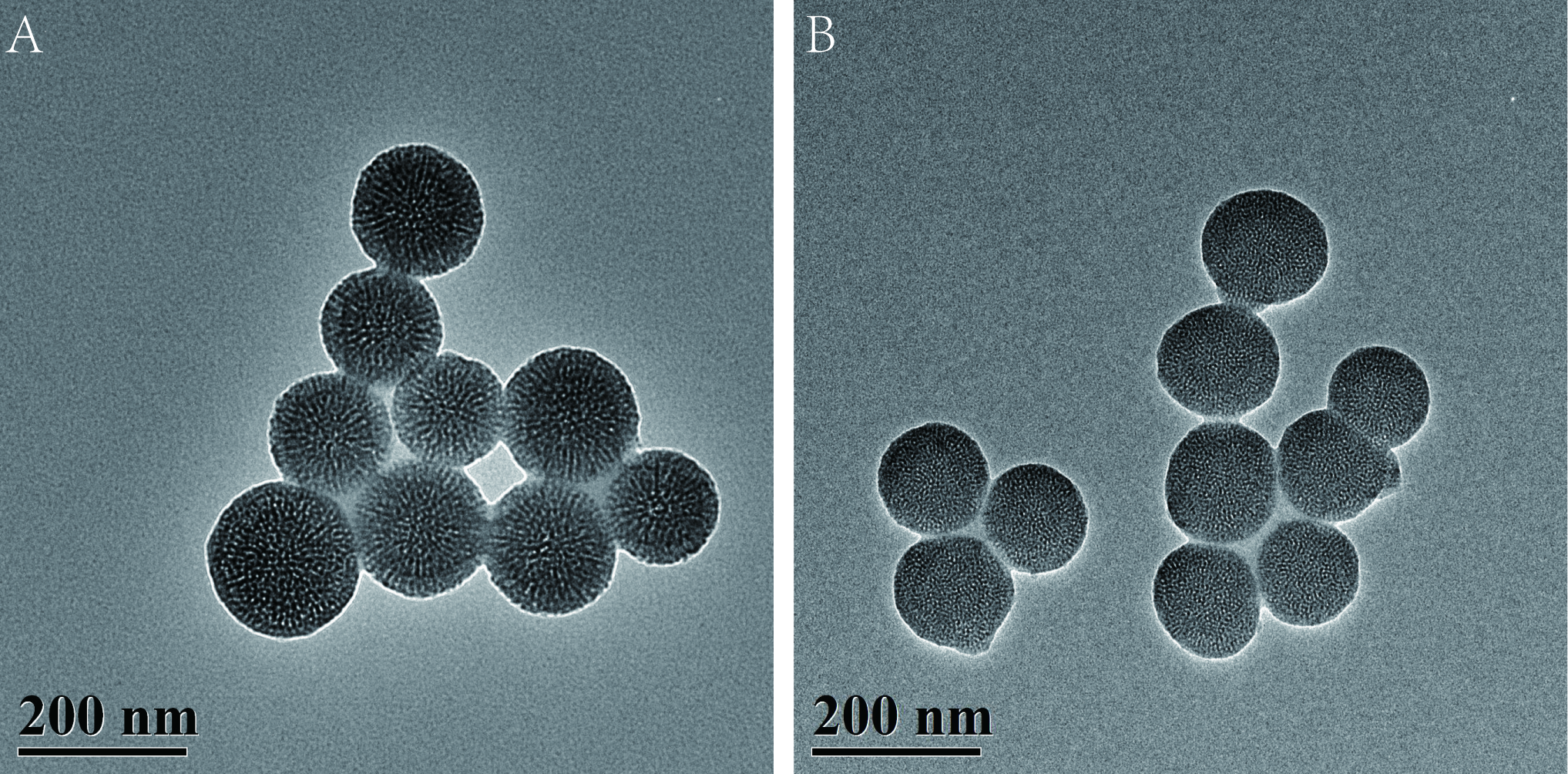


**Fig.S3** TEM images of (A) E/DMSN_1_ and (B) E/DMSN_2_.

**Table S1** Data from TGA (percentage weight loss) and from UPLC analysis (LC%)

| Samples | TGA | UPLC |
| --- | --- | --- |
| E/DMSN_1_ | 53.08% | 55.55% |
| E/DMSN_2_ | 46.66% | 42.76% |

**Table S2** Textural properties of the silica-based samples

| Samples | Partical size (nM) | Zeta potential (mV) | Pore size (nM) | SSA (m^2^ g^-1^) | Pore volume (cm^3^ g^-1^) |
| --- | --- | --- | --- | --- | --- |
| DMSN_1_ | 164 | -27.6 | 3.5 | 274 | 0.289 |
| DMSN_2_ | 161 | -30.3 | 4.6 | 460 | 0.523 |
| E/DMSN_1_ | 225 | -25.2 | 2.6 | 24 | 0.057 |
| E/DMSN_2_ | 207 | -26.5 | 3.2 | 7 | 0.014 |


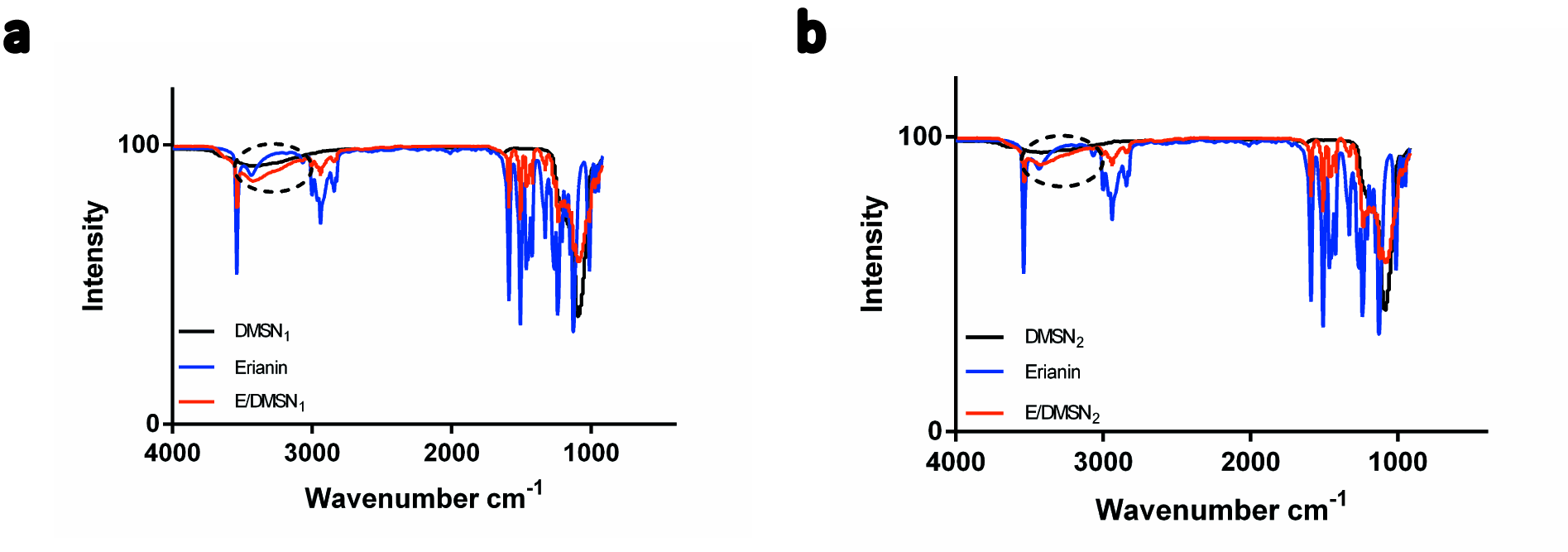


**Fig. S4** FITR spectra of DMSN_1_, erian, and E/DMSN_1_ in (A) and of DMSN_2_, erian, and E/DMSN_2_ in (B).





**Fig. S5** The chemical structure of erianin.


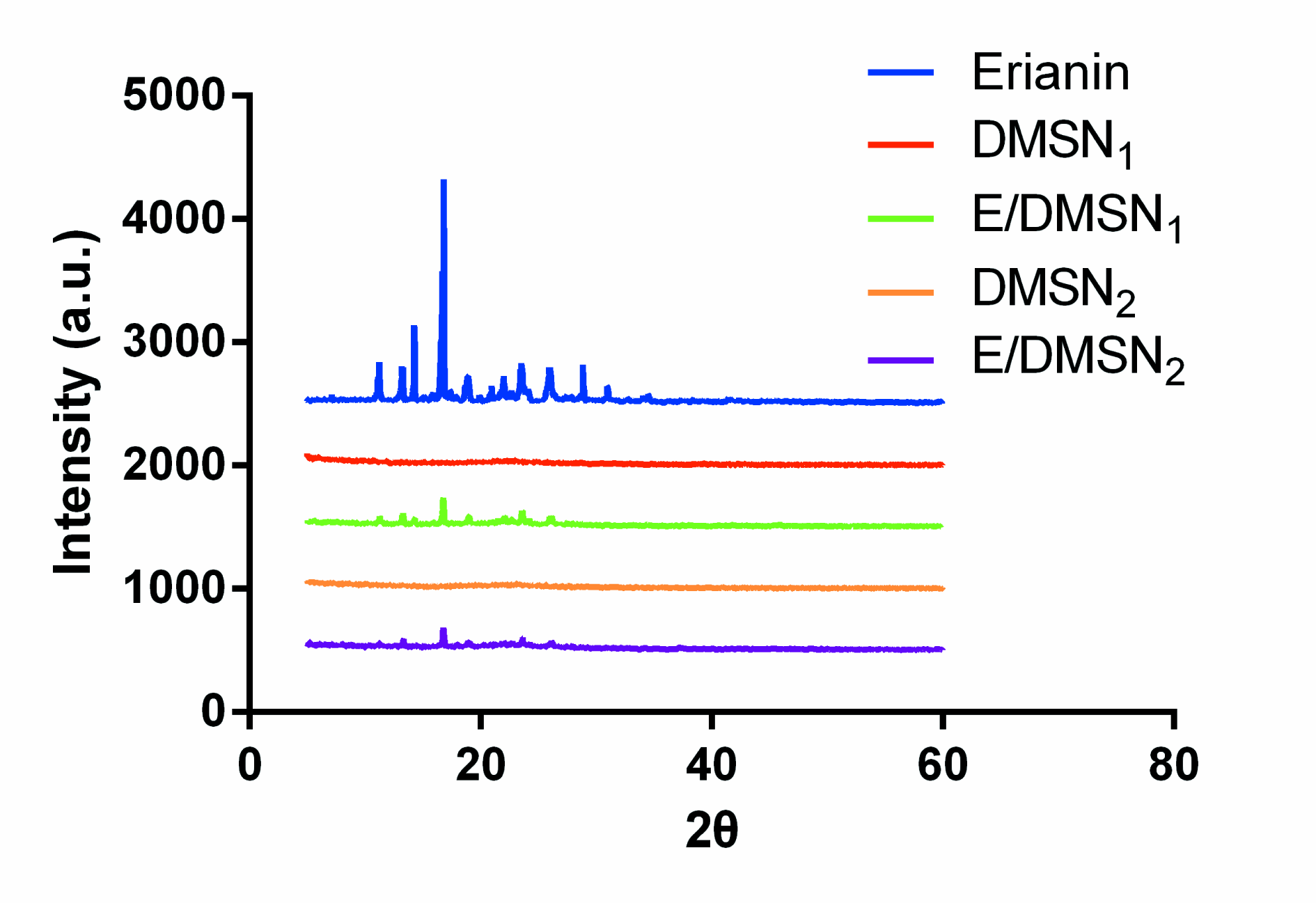


**Fig. S6** wide angle XRD analysis of all samples.


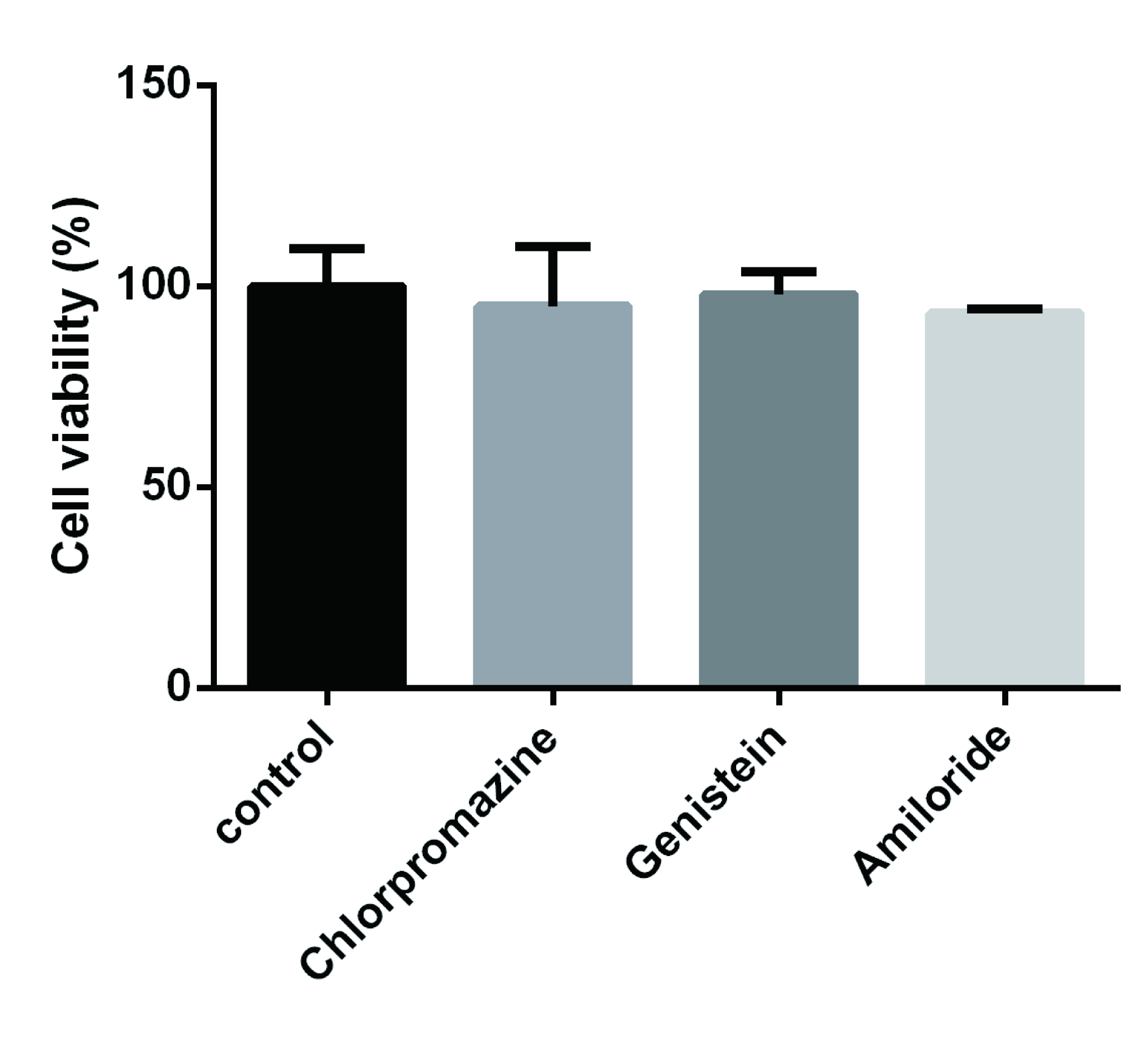


**Fig. S7** Cytotoxicity evaluation of three inhibitors in HaCaT cells for 24 h.


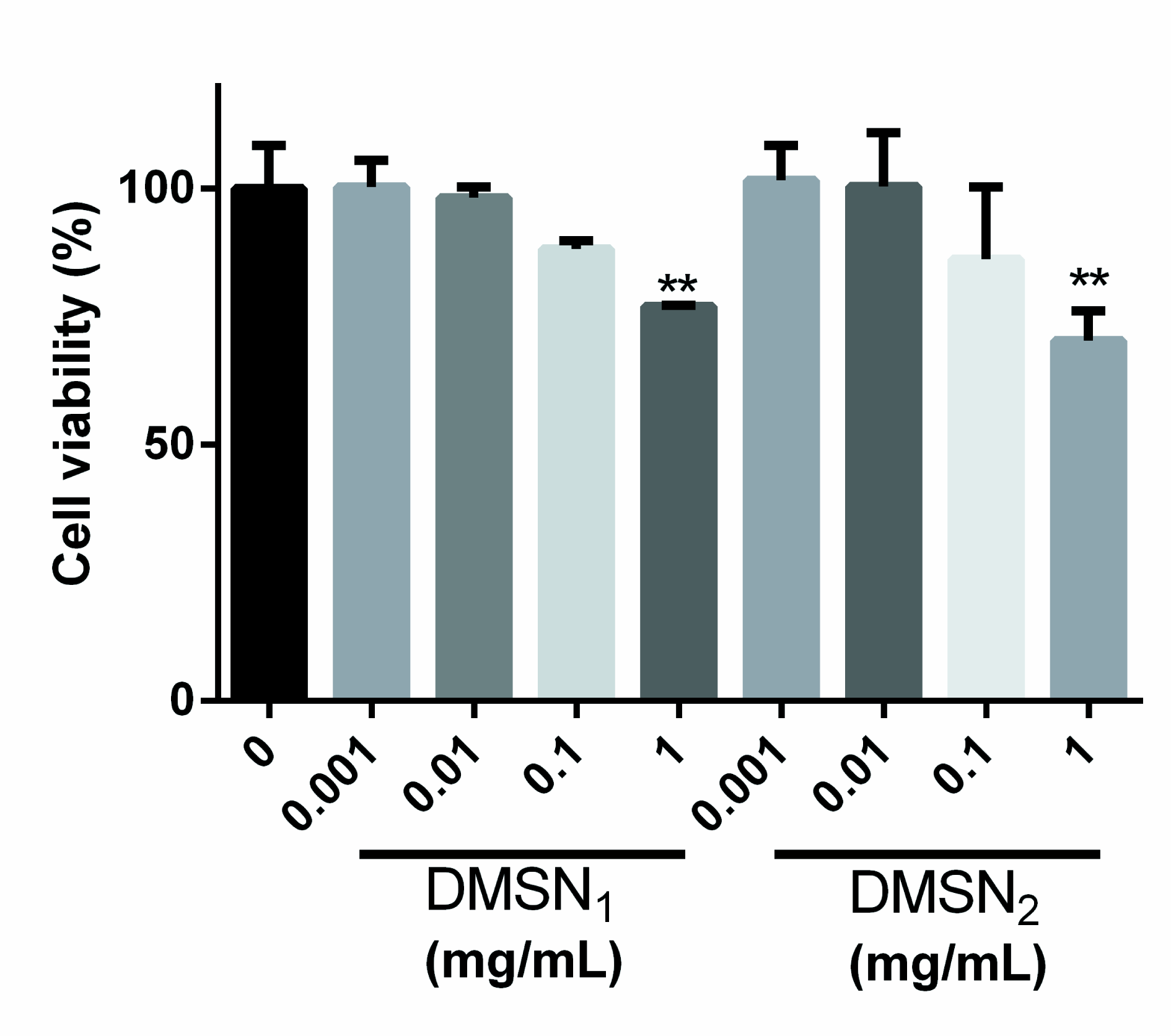


**Fig. S8** Cytotoxicity evaluation of DMSNs in HaCaT cells for 24 h.
